# HIV-Tat protein-accelerated aging

**DOI:** 10.18632/aging.204105

**Published:** 2022-05-27

**Authors:** Marc J. Kaufman, Alaa N. Qrareya, Jason J. Paris

**Affiliations:** 1 McLean Imaging Center, McLean Hospital, Harvard Medical School, Belmont, MA 02478, USA; 2 Department of BioMolecular Sciences, School of Pharmacy, University of Mississippi, University, MS 38677, USA

**Keywords:** aging, BDNF, HIV, nanotechnology, Tat, taurine

Human Immunodeficiency virus (HIV) infection accelerates aging [1]. This effect is expected to become increasingly problematic because currently, in the United States, more than half of those infected with HIV are over 50 years old and thus are subjected to the combined effects of HIV and aging [1]. Antiretroviral therapy (ART) for HIV greatly suppresses viral loads and reduces HIV-related morbidity and mortality, enabling people living with HIV (PLWH) to have a much better quality-of-life and survive into old age. However, ART does not prevent neurological disturbances from developing in PLWH including disruptions in mood and cognitive ability and the development of intractable pain states. These comorbidities reduce quality-of-life and catalyze problematic behaviors including substance use disorders that worsen HIV- and age-related outcomes. These sequelae occur in part because ART does not stop HIV reservoirs from synthesizing and releasing toxic proteins such as the transactivator of transcription (Tat) protein, appreciable levels of which are present in the central nervous system during ART (e.g., [2]). In particular, Tat is neurotoxic and it induces neuroinflammation, oxidative stress, and mitochondrial dysfunction (see [3]), effects that also are associated with aging [1].

Although HIV is a complicated disease involving accumulations of several toxic viral proteins, given Tat’s neurotoxicity and its continued expression during ART, researchers have developed several transgenic mouse lines that selectively express Tat protein (e.g., iTat mice) to better assess Tat’s mechanistic effects and to evaluate novel interventions. Studies in iTat mice support the theory that Tat contributes to the mood-related, cognitive, behavioral, and brain structural and functional abnormalities observed in PLWH, and that Tat, like HIV, accelerates age-related pathophysiology (e.g., [3–5]). These studies also support the possibility that two novel treatments, a low-tech taurine supplementation treatment [3] and a high-tech bioengineered brain derived neurotrophic factor (BDNF)-nanoparticle treatment [5], may attenuate the separate and interacting effects of HIV and aging.

Our high magnetic field (9.4 Telsa) magnetic resonance spectroscopy (MRS) study in aged transgenic iTat mice, which experience prolonged Tat transgene leak that in some ways models Tat release from human HIV reservoirs, detected abnormally low brain taurine levels [3]. Taurine attenuates inflammation, oxidative stress, neurotoxicity, and mitochondrial dysfunction, and thus low taurine levels could be permissive to these effects induced by Tat, by HIV, and by aging (see [3]). Low taurine levels can be mitigated by dietary taurine supplementation, which when combined with exercise, reduced inflammation and improved cognition among elderly women [6]. In aged mice, taurine supplementation induced hippocampal neurogenesis [7], an effect that could improve cognition. Given these beneficial effects, taurine’s low cost and high safety profile, and its ability to oppose potentially convergent effects of aging and Tat or HIV, it seems that taurine supplementation should be tested in animal models of HIV and in PLWH. Such a study would be innovative as to date, taurine supplementation has not been tested in any HIV model. Human brain taurine deficiencies have not yet been reported in HIV patients or in the elderly, perhaps because MRS quantification of taurine is difficult at magnetic field strengths typically used in human studies (≤4 Tesla). Thus, it may be necessary to use higher magnetic field strengths (e.g., 7 Tesla) to test whether brain taurine levels are depleted in PLWH and in the elderly.

Regarding the high-tech nanoparticle treatment, a recently published study reported that intranasal BDNF-nanoparticle administration substantially increased hippocampal BDNF protein levels in young adult iTat mice induced with doxycycline to express Tat, which by itself lowers BDNF levels [5]. BDNF-nanoparticles also improved learning and memory and increased neurogenesis and synaptogenesis, each of which is impaired by Tat and by HIV ([5] and references therein). Older mice were not tested in this study, but it is plausible that BDNF-nanoparticle treatment also could benefit older subjects.

BDNF-nanoparticles are engineered on a clathrin triskelion backbone containing up to 3 functional groups [5]. Thus, in addition to BDNF (or some other therapeutic moiety), a clathrin nanoparticle could be engineered to contain an affinity ligand that enables selective targeting and/or a reporter ligand that enables nanoparticle localization with imaging technology. Thus, this technology is flexible and has theranostic potential.

Interestingly, taurine and BDNF-nanoparticles may exert some common effects as in aged mice, taurine treatment induced hippocampal neurogenesis [7] and in diabetic rats, taurine tripled hippocampal BDNF mRNA levels and concurrently improved short-term memory [8]. However, a finding of increased BDNF mRNA levels does not necessarily reflect increased mature (mBDNF) protein levels. In this regard, BDNF mRNA is translated to make pro-BDNF protein, the mBDNF precursor, and additional enzymatic processing is needed to convert pro-BDNF to mBDNF. This last step, if deficient, could result in an elevated pro-BDNF/mBDNF ratio, an effect that can oppose mBDNF signaling (see [5]). Thus, it remains to be determined whether taurine’s beneficial effects on memory are mediated by increased mBDNF protein levels. Given the possibility that taurine and BDNF-nanoparticles may exert parallel and convergent effects, it may be worth testing different taurine/BDNF-nanoparticle combinations to see whether their combined effects are greater than either treatment alone.

Together, these studies illustrate 1) the utility of transgenic mouse models to selectively study effects of HIV-related proteins, 2) how Tat protein effects could contribute to the combined effects of aging and HIV, 3) that novel therapeutic approaches may benefit older people, including PLWH, by targeting common pathophysiological effects of aging and HIV, and 4) highlight some literature gaps worth filling.
